# Bone response in vivo of Ti-45Zr alloy as dental implant material

**DOI:** 10.1007/s10856-022-06664-5

**Published:** 2022-05-21

**Authors:** Pinghua Ou, Taomei Zhang, Jianying Wang, Cui Li, Chunsheng Shao, Jianming Ruan

**Affiliations:** 1 Department of Stomatology, The Third Xiangya Hospital, Central South University, 410013 Changsha, Hunan PR China; 2 State Key Laboratory of Powder Metallurgy, Central South University, 410083 Changsha, Hunan PR China

## Abstract

Ti-Zr alloys have gained increasing attention as a new metallic biomaterial, being used as implants for both orthopedics and dentistry. More recently, our group found promising results for the Ti-45Zr alloy, which presented a low elastic modulus, a pronounced and excellent mechanic character, and excellent cell compatibility in vitro. However, its biocompatibility and potential to promote osteogenesis in vivo remained unclear. In the present study, the biocompatibility, osteointegration ability, and immune response effects of the Ti-45Zr alloy were evaluated in animal experiments. The results showed that the alloy had good blood compatibility and no body side effects. After implantation in vivo, the inflammation turned out well and was beneficial to the polarization of macrophages. Additionally, the Ti-45Zr alloy presented a good osteointegration ability. Overall, these results confirmed that the Ti-45Zr alloy can be used as a dental implant material.

## Introduction

Dental defects are common oral diseases. They can affect the chewing function of patients, leading to alveolar bone and masticatory muscle atrophies, facial collapse, and other problems. Complete dentition loss can lead to patients suffering from malnutrition syndrome, which will affect their quality of life. Thus, tooth loss repair is particularly important in oral treatment. Dental implant prostheses have become a popular treatment for those defects, due to a high success rate, good predictability, and fewer complications [1].

At present, the Ti-6Al-4V alloy is widely used as implant material in the clinic due to its good corrosion resistance and high mechanical strength. However, this alloy contains cytotoxic elements (such as Al and V) that can cause adverse health problems when released into the body [2]. Additionally, the elastic modulus of the Ti-6Al-4V alloy (112 GPa) is higher than that of human bone tissue (0.02–2 GPa of cancellous bone and 7.7–21.8 GPa of cortical bone) [3, 4], which might lead to “stress shielding” effects, then cause bone tissue absorption, implant loosening, and even implant shedding [5]. Therefore, the development of dental implant materials with non-toxic side effects, excellent biocompatibility, elastic modulus adaptation, and good osseointegration is of great importance.

Furthermore, zirconium (Zr) is a “biophile” metal that has good biocompatibility and excellent corrosion resistance [6, 7]. Meanwhile, as a transition metal, it can form a solid solution with other metallic elements (Ti, Nb, and Ta). The Ti-Zr alloy can improve the mechanical properties of titanium (such as compressive strength, hardness, and flexure strength) and enhance wear resistance [8]. Moreover, the Ti-Zr alloy has excellent biocompatibility and corrosion resistance [9–11]. In our previous study, a Ti-45Zr alloy was successfully prepared by traditional powder metallurgy, and its mechanical and biological properties were systematically evaluated. The results showed that the alloy exhibited good biomechanical properties and excellent cytocompatibility [12]. However, biocompatibility and osseointegration remained to be further studied in vivo.

Biocompatibility refers to the normal function of a material in the body. The ideal biomedical implant material must be non-toxic and not cause an allergic reaction in the body. At the same time, osseointegration is the main criterion of dental implant success [13]. Hence, it is essential to evaluate the biocompatibility and osseointegration of a material. Frequently, in vivo biocompatibility studies are performed by animal implantation. The use of animal models is an important step for testing dental implant materials before clinical application in humans. Implantation experiments in animals can mimic the internal environment to determine the optimal interface between the bone and the implanted material. Animal implantation tests are a pre-clinical test for biocompatible materials, and mainly detect toxic reactions, immune responses, and osteointegration ability in vivo.

In the present study, the biocompatibility and osseointegration of the Ti-45Zr alloy were systematically studied in animal implantation experiments, using enzyme-linked immunosorbent assay (ELISA), hard tissue sections hematoxylin-eosin (HE) staining, immunohistochemical (IHC) staining, and hemolysis test to evaluate its toxic and side effects, immune responses, and osteointegration ability. Overall, we provided the theoretical basis for the clinical application of the Ti-45Zr alloy. The experiments involving animals were approved by the animal care and use ethical committee of Central South University (No: 2019syuw0225) and Hunan Academy of Traditional Chinese Medicine (No: 2021-0023), and complied with the Guide for the Care and Use of Laboratory Animals approved by the National Institutes of Health.

## Materials and methods

### Sample preparation

The Ti-45Zr alloy was provided by the Powder Metallurgy Research Institute of Central South University. The Ti-45Zr alloy and cpTi were prepared in a cylinder (Ф4 mm × 6 mm, Ф1.5 mm × 4 mm) and a circular metal sheet (diameter of 10 mm and thickness of 1 mm) by a mechanical cutting method. Then, the samples were mechanically polished with #100, #200, #800, and #1200 silicon carbide sandpaper, and cleaned by ultrasonic wave with acetone, ethanol, and deionized water for 15 min each. After cleaning, samples were sterilized by a vacuum high-temperature disinfection machine (Vacuklav24B/L+, Germany). After drying, samples were aseptically packaged. According to the experimental design, cylindrical samples (Ф4 mm × 6 mm) were used for the implantation experiment in rabbits. The other cylindrical samples (Ф1.5 mm × 4 mm) were used for the implantation experiment in rats, and the round metal pieces (Ф10 mm × 1 mm) were used for the hemolysis experiment.

### Microstructural characterization

The microstructural features of the Ti-45Zr alloy were characterized using electron microscopes. Scanning electron microscope (SEM, FEI Helios NanoLab G3 UC) equipped with energy dispersive spectrometry (EDS, Team Octane Plus), electron probe microanalysis (EPMA, JXA-8530F), and transmission electron microscope (TEM, JEM-2100F). The TEM samples were prepared using the precision ion polishing system (PIPS) at a voltage of 5 kV/2 kV and an incident angle of 3–7°.

### Hemolysis test

Blood from healthy volunteers (20 mL) was extracted and placed in a centrifuge tube (50 mL), and 1 mL of freshly prepared 2% potassium oxalate normal saline solution was added for anticoagulation. The anticoagulant human blood and normal saline (0.9%) were mixed in a 4:5 ratio (diluted blood) and stored at 4 °C for later use. After polishing and ultrasonic cleaning, the metal sheet samples (Ф10 mm × 1 mm) were placed in a 15 mL centrifuge tube and 10 mL of normal saline (0.9%) was added (experimental group). The negative control group consisted of 10 mL of normal saline (0.9%) without a metal sheet and the positive control group of 10 mL of distilled water without a metal sheet. Centrifuge tubes were placed in a 37 °C thermostatic water tank for preheating for 30 min. Diluted blood (0.2 mL) was added to each centrifuge tube and kept for 60 min in a 37 °C thermostatic water tank. Then, each centrifuge tube was removed and centrifuged (3000 rpm/5 min). The supernatant was placed in a colorimetric cup, and the absorbance was measured at 545 nm in the spectrophotometer. The experiment was repeated three times for each group.

### Immune responses in vivo

#### Surgical method

**Fig. 1:**
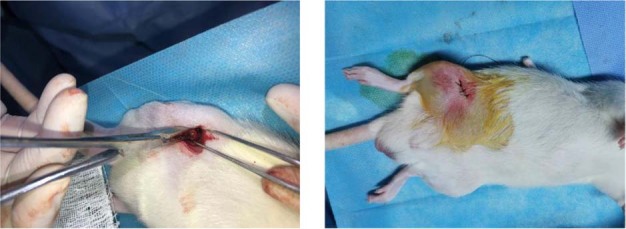
Rats implantation surgery

Sprague Dawley (SD) rats (12 w, 350 ± 30 g) were used as animal models. After a two-week acclimation environment, the rats were randomly assigned to two groups (*n* = 9/group). Before implantation, 10% chloral hydrate solution (0.3 mL/100 g) was intraperitoneally injected for anesthesia. After the rats entered the anesthetic state, hair removal and disinfection were performed in the operative area. The femur middle segment surface skin was cut open, subcutaneous tissue and muscle were separated, and the medial femur was exposed. Then, the periosteum was removed with a periosteum stripper to fully expose the visual field. A cylindrical hole (Ф1.5 mm × 4 mm) was prepared on the femur’s right middle segment vertical surface. Next, the implant material (Ф1.5 mm × 4 mm) was placed into the hole, the wound was washed with normal saline, and the incision was layered sutured (Fig. 1). On the first day after surgery, all rats received a 0.3 mL Cefepime (0.02 g/mL) intramuscular injection to prevent infection. On postoperative days 3, 9, and 14, rats were sacrificed by chloral hydrate anesthetic injection. The wound healing was observed and rats’ femurs were dissected, removed, and fixed in 4% paraformaldehyde.

#### ELISA

Fresh blood was collected from the experimental and control groups at three time points (days 3, 9, and 14) and serum was collected for ELISA. Rat’s interleukin-6 (IL-6) and interleukin-10 (IL-10) kits (Jiangsu Jingmei) were used according to the manufacturer’s instructions. The serum Optical Density (OD) was measured at 450 nm. A standard curve was prepared to calculate the concentration of inflammatory factors. Then, the levels of IL-6 and IL-10 in serum were determined.

#### Hard tissue sections HE staining

After removal of the implant material, the femoral bone segments were soaked in 4% paraformaldehyde for 24 h, then immersed in 15% EDTA solution for continuous decalcification (30 d). The solution was changed when necessary. After complete decalcification, samples were rinsed with water for 6 h. The bone tissue was repaired with a scalpel, and a 4 mm bone segment with the implant hole was taken as the sample. Then, samples were dehydrated (automatic dehydrator), embedded in paraffin, and sectioned (about 7 μm thick). Sections were stained with HE, and images were collected using an Olympus BX50 biological microscope.

#### IHC staining

IHC staining of macrophage surface markers was performed on the decalcified sections. Dewaxed sections were rinsed with PBS 3 times, 3 min each, then 1% trypsin was dropped to cover the tissue and placed in an incubator at 37 °C for 20 min for repair. Next, sections were repeatedly washed with water and PBS and placed in a 0.3% hydrogen peroxide aqueous solution for 10 min to remove endogenous peroxidase. Samples were rinsed with PBS 3 times, 3 min each. The primary antibody was added at 4 °C overnight. The second antibody was dropped and placed in an incubator at 37 °C for 20 min. Samples were analyzed using the DAB color kit. Briefly, the reagents were mixed and dropped onto the slices, then colored at room temperature for 5 min away from light and washed with distilled water. Mild redyeing of hematoxylin was performed for 3 min, followed by dehydration, xylene transparent for 1 min, neutral gum sealing sheet, and image collection. Each section was observed at 100x magnification, and 5 field images were randomly selected at 400x magnification. The image analysis system Image-Pro Plus 6.0 was used to measure the optical density (IOD) and image’s dyed area. The mean density of each image was calculated and the average value of 5 images was considered as the experimental result. Additionally, the number of cells with antigen 68 (CD68) positive (CD68^+^), inducible nitric oxide synthase (iNOS) positive (iNOS^+^), and arginase-1 (Arg1) positive (Arg1^+^) differentiation around the implant cluster was counted at 400 fold. By calculating the ratio of iNOS^+^ and Arg1^+^ cells to CD68^+^ cells, the M1 and M2 types polarization ratio of macrophages was evaluated. The primary antibodies were CD68, iNOS2 and Arg1. On days 3, 9, and 14, samples were IHC stained with CD68, iNOS2, and Arg1 to analyze the early post-implantation inflammatory response and macrophages’ biological behavior.

### Osseointegration in vivo

#### Surgical method

**Fig. 2:**
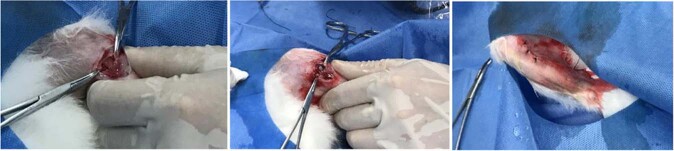
Rabbits implantation surgery

New Zealand male white rabbits (NZW, 12 w, 2.5 ± 0.5 kg), purchased from the Experimental Animal Center of Central South University, were selected to evaluate the alloy’s biosecurity in vivo. The rabbits were randomly assigned to the Ti-45Zr or cpTi groups (*n* = 6/group) for the implantation experiment. After two weeks of domestication, feeding, and environmental adaptation, a 3% pentobarbital solution (2 mL/kg) was intravenously injected into the ear margin to put animals under general anesthesia. Then, 2% lidocaine was injected into the surgical site for local infiltration into the periosteum anesthesia. A longitudinal incision (2 cm) was made on the knee joint medial side to cut the skin and muscle, and the deep tissue was separated by a bland method to expose the femoral head medial side. Then, the periosteum was removed with a periosteum dissection device to fully expose the visual field. The rotary speed of the implant was adjusted to 1000 rpm. Drilling was carried out at a slow speed on the vertical bone surface inside the femoral head, and the drilling process was cooled with sterile normal saline. Bone fragments were removed by washing, and the holes were gradually expanded at a constant speed to prepare for hole formation (6 mm depth and 4 mm diameter). Finally, the metal implant material (Ф4 mm × 6 mm) was placed into the hole so that its top was flush with the bone surface. The wound was repeatedly washed with normal saline, and the periosteum, muscle, and skin were stratified and sutured (Fig. 2). Postoperatively, 40 mg/kg of ceftiofur was intraperitoneally injected to prevent wound infection. When animals woke up and moved on their own, they were sent back to the feeding center. They were normally fed in a single cage, and their affected limbs were allowed to move freely with weight. Within 5 days after the operation, the rabbits were daily injected with 40 mg/kg antibiotics into their buttocks to prevent infection, and the wounds were daily checked to ensure cleanliness. According to the experimental design, animals were killed by air embolization at the 12th and 16th weeks after surgery. The femoral end with the implant was dissected, and the bone segment with the implant material, as well as the organs (liver, kidney, and heart) were removed and quickly stored in a 4% neutral buffered formalin solution for subsequent experiments.

#### Gross sample observation

Animals were killed according to the predetermined time point of the experimental design, and anatomical materials were immediately collected. During the anatomical surgery process, incision healing at the implantation site, the soft tissue around the implant, and the combination of the implant and bone tissue were observed. The femur with the implant material was removed, labeled, numbered, and photographed.

#### Imaging evaluation

The femur’s anterior and lateral films (12 and 16 w) with implant materials were taken by an X-ray machine (Third Xiangya Hospital of Central South University) to observe the interface between the implant and the surrounding bone tissue. The radiographs were observed and analyzed for the presence of low-density images and to observe new bone formation around the implant material. If the low-density area was greater than 0.5 mm, it indicated bone tissue absorption around the implant.

#### Hard tissue observation by grinding and staining

The femur with the implant material was immersed in a 4% neutral buffered formalin solution. After 48 h, the femur was rinsed with water, dehydrated in a graded ethanol series (70, 80, 90, and 95%), and soaked in different concentrations of Technovit7200 resin solution (the solvent was anhydrous ethanol). Then, samples were embedded in resin and polymerized by a light-curing machine. The tissue mass was removed and sliced along the implant material’s long axis using a German Exakt-300CP hard tissue slicer. The tissue was cut into 200 μm thick slices and grounded to 25 μm with 300#, 800#, 1200#, and 2000# sandpaper using a German Exakt400S grinding machine. Cut marks were removed by polishing with 4000 mesh sandpaper. Non-decalcified slices (20 μm thick) were retained and pasted on 0.2 cm glass slides. The slices were stained with 1% methylene blue solution for 10 min, washed with deionized water to remove dye excess, stained with 2% alkaline magenta for 5 min, washed with 95 and 100% alcohol for 10 min each, treated with xylene transparent, and finally sealed. The section’s tissue morphology was observed under a biological microscope.

### Statistical analyses

The experimental data are expressed as means ± standard deviations (SD). An *n* = 5 specimens/group was used for immunohistochemical quantitative analysis and *n* = 3 specimens/group was used for the rest of the analyses. The t-tests were performed using GraphPad Prism 6 software. A *p* < 0.05 was considered statistically significant.

## Results

### Structure of the Ti-45Zr alloy

**Fig. 3:**
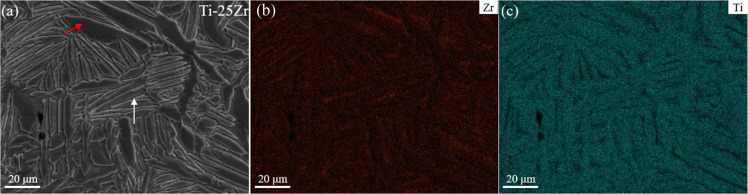
**a** Backscattered SEM micrograph showing the microstructure of the Ti-45Zr alloy (red arrow for the dark lamellae phase, white arrow for the white needle phase); SEM/EDS mapping showing the elemental distribution, including (**b**) Zr and (**c**) Ti

**Fig. 4:**
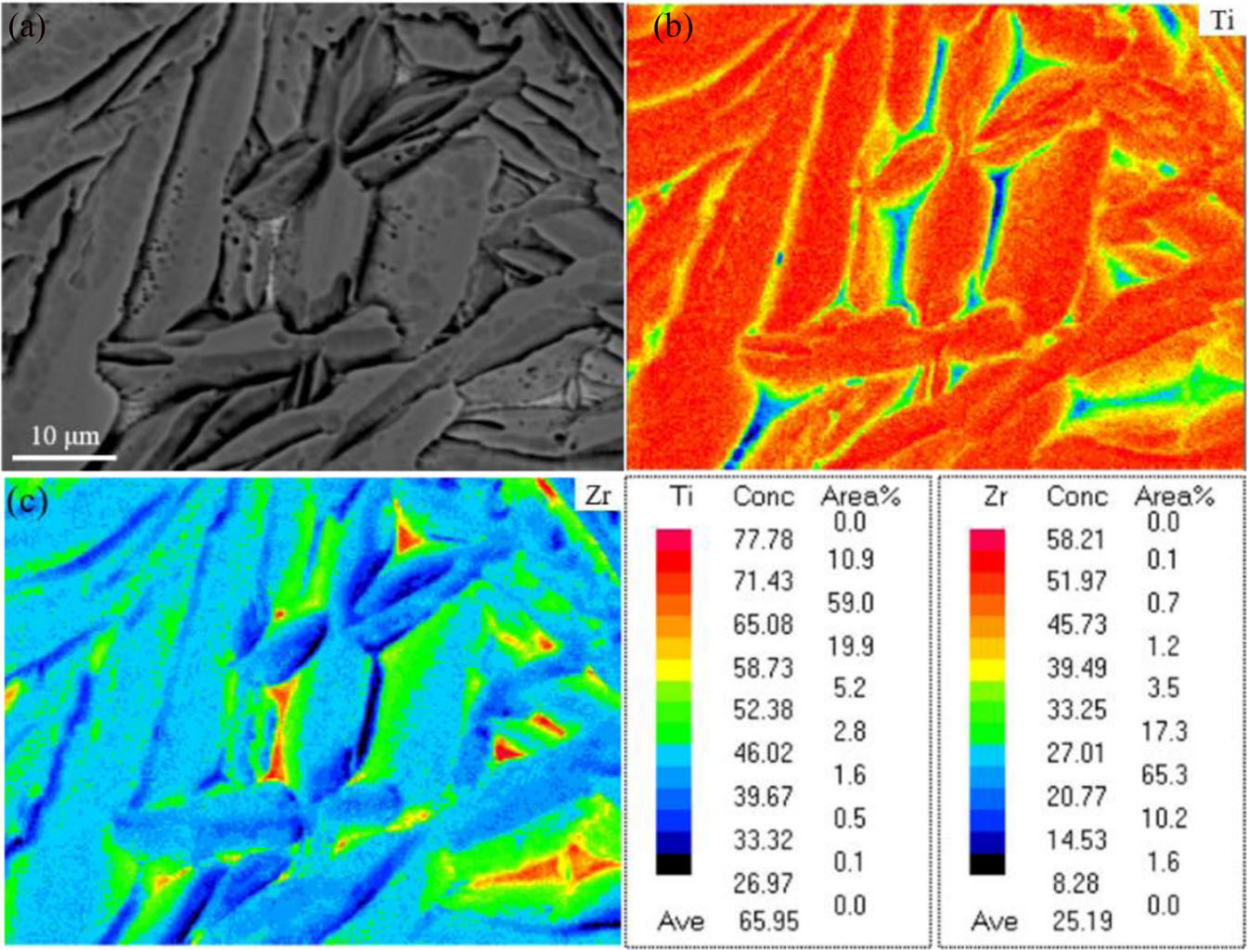
EPMA element scanning diagram of the Ti-45Zr alloy: (**a**) Ti-45Zr; (**b**) Ti and (**c**) Zr

The SEM micrograph in Fig. 3a shows the general microstructure of the Ti-45Zr alloy with a few pores. Two different phases were found in the microstructure: (i) α phase: dark lamellae phase with a relatively large width; (ii) α′ phase: white needle phase with a relatively small width. Elemental mapping was carried out by SEM/EDS (Team Octane Plus). The α phases were enriched in Ti, while the α′ phase was enriched in Zr (Fig. 3b, c). The EPMA was further performed to characterize the elemental distributions in the Ti-45Zr alloy (Fig. 4). Similarly, Ti was uniformly distributed in the α phase, while Zr was mainly distributed in the α′ phase.

**Fig. 5:**
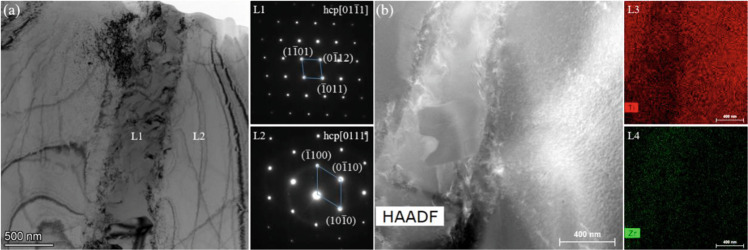
Bright-field image and selected electron diffraction pattern area of the Ti-45Zr alloy: (**a**, **b**) bright-field image; diffraction pattern for the L1-L2 area; (L3-L4) HAADF diagram and corresponding transmission element plane distribution diagram

The TEM images, the corresponding selected area electron diffractions patterns (SADPs), and the elemental distribution of the Ti-45Zr alloy are presented in Fig. 5. The α phases with hcp (hexagonal close-packed) structure were enriched in Ti. Meanwhile, the α′ phases with hcp structure were enriched in Zr. These results were consistent with the SEM observation.

### Blood compatibility of the Ti-45Zr alloy

The blood compatibility of a biomaterial is one of the most important criteria to determine whether the material can be successfully applied in clinical practice. If the blood compatibility of the implant material is poor, a series of reactions, such as hemolysis, coagulation, and immune rejection, can occur in the body, leading to implant failure. Therefore, a hemolysis test was used to evaluate the blood compatibility of the Ti-45Zr alloy. The hemolysis rate was calculated according to Equation 1–1 and the results are shown in Table 1. The hemolysis rate of the Ti-45Zr alloy was 1.18% and followed national standards (<5%). Therefore, the Ti-45Zr alloy had low hemolysis and good blood compatibility.

1-1 $$\begin{array}{l}{{{\mathrm{Hemolysis}}}}\,{{{\mathrm{ratio}}}} =\\ \left[ \begin{array}{l}\left( {{{{\mathrm{Experimental}}}}\,{{{\mathrm{group}}}}\,{{{\mathrm{OD}}}} - {{{\mathrm{Negative}}}}\,{{{\mathrm{control}}}}\,{{{\mathrm{group}}}}\,{{{\mathrm{OD}}}}} \right)/\\ \left( {{{{\mathrm{Positive}}}}\,{{{\mathrm{control}}}}\,{{{\mathrm{group}}}}\,{{{\mathrm{OD}}}} - {{{\mathrm{Negative}}}}\,{{{\mathrm{control}}}}\,{{{\mathrm{group}}}}\,{{{\mathrm{OD}}}}} \right)\end{array} \right] \ast 100\%\end{array}$$

**Table 1:** Hemolysis rate of the Ti-45Zr alloy

Group	OD value			Mean value	Hemolysis ratio
	1	2	3		
Ti-45Zr	0.038	0.035	0.032	0.038	1.18%
Negative	0.024	0.025	0.025	0.0243	
Positive	0.942	0.913	0.866	0.907	

### Osteo-immunomodulation in vivo

#### Secretion of inflammatory factors

**Fig. 6:**
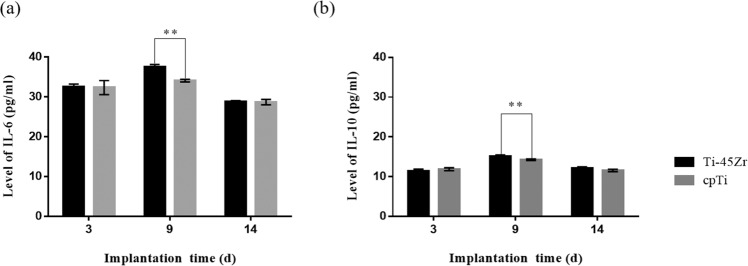
Serum concentrations of inflammation-related factors: (**a**) IL-6; (**b**) IL-10. **p < 0.01

Macrophages are the main inflammatory immunomodulatory cells that regulate and terminate the inflammatory response by secreting various inflammatory factors. IL-6 and IL-10 are major pro-inflammatory and anti-inflammatory factors in this response, playing an important role in implantation processes and outcomes. In the present study, the IL-6 and IL-10 in the rats’ blood were detected by ELISA at different time points (Fig. 6). In general, IL-6 concentrations reached the highest level on the 9th day and were significantly downregulated on the 14th day. The IL-10 concentration in the Ti-45Zr group reached the highest level on the 9th day and was downregulated on the 14th day. Compared to the cpTi group, the levels of IL-6 and IL-10 were significantly higher in the Ti-45Zr implant group on the 9th-day post-surgery (*p* < 0.01).

#### Inflammatory response after implantation

**Fig. 7:**
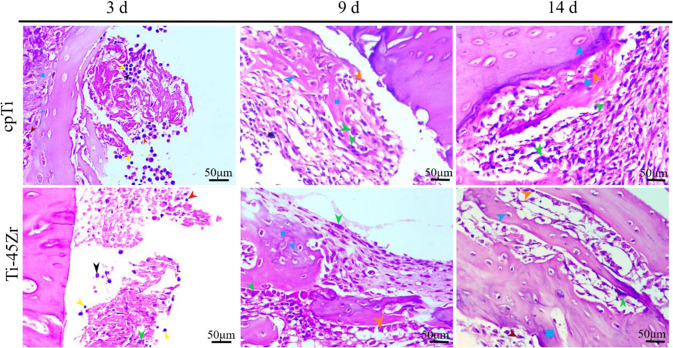
HE staining histological observation of rats: neutrophils (red arrows); macrophages (green arrows); lymphocytes (yellow arrows); monocytes (black arrows); osteoblasts (orange arrows); osteoclasts (brown arrow); chondrocytes (blue arrows); fibrous tissue (gray oval); bone like matrix (blue square)

Implantation can trigger the body’s immune defense mechanism. The most typical response is implanting inflammation. Moreover, the inflammatory response outcome regarding the implant stability and bone formation ability is very important. In the present study, the inflammatory response around the implant material was observed by HE staining. On the 3rd day, the main pathological change in each group was inflammatory exudation (Fig. 7). The main inflammatory cells were neutrophils and a small number of lymphocytes and monocytes was also observed. No clear pathological changes were observed in the bone tissue around the cave implantation. On the 9th day, few osteoid matrices and osteoblasts were detected in the inflammatory foci and the main inflammatory cells were monocytes and macrophages. The number of macrophages increased compared with the 3rd day, and a few multinucleated macrophages were observed. Around the 14th day, fibrous connective tissue hyperplasia formed inside the implantation hole. Interstitial mononuclear cell infiltration was visible in the trabecular bone structure, bone matrix, cartilage cells, and osteoblasts. New bone formation occurred in the later inflammatory response stage and the prognosis was good.

#### Infiltration of macrophages

**Fig. 8:**
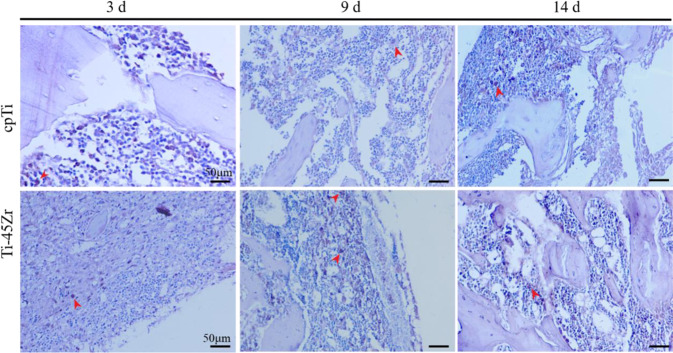
CD68 immunohistochemical staining and histological observation of rats at 3, 9, and 14 d after the Ti-45Zr alloy and cpTi implantation (red arrow indicates positive color area)

**Fig. 9:**
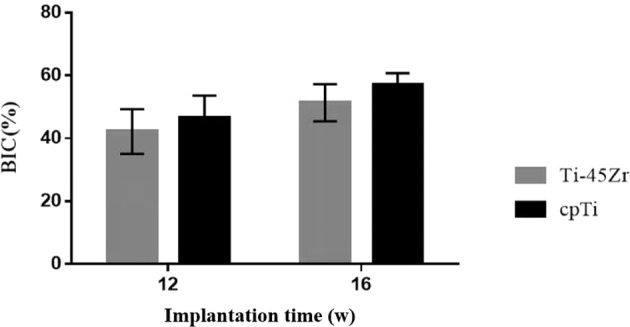
Mean density of the CD68 positive fraction at 3, 9, and 14 days after implantation. **p < 0.01

Macrophages are the first cells that contact the material and participate in immune regulation. In the inflammatory response stage, macrophages will arrive at the material’s surface mediated by chemokines and participate in the regulation of the inflammatory response. The early infiltration of macrophages contributes to the initial inflammatory response and debridement at the implant site. Here, we used IHC staining to detect the expression of the specific marker CD68 on the surface of macrophages, allowing the evaluation of the distribution of their infiltration around the implanted material. Macrophages with brown nuclei were deeply stained, and the red arrow indicates the CD68 positive staining area (Fig. 8). On the 9th day, the positive staining area increased compared to the 3rd and 14th days. Also, on the 9th day, the infiltration of macrophages around the implanted hole increased, consistent with the HE staining results. The optical density of the staining area was measured by Image-Pro Plus 6.0 Image analysis system, and the average for each group was calculated. On the 9th day, the average optical density of the CD68 positive region was significantly increased compared to the 3rd day. On the 14th day, the average optical density of the CD68 positive region decreased. Furthermore, the mean density of the CD68 positive region was markedly higher in the Ti-45Zr implant group compared to the cpTi group on the 14th day after surgery (*p* < 0.01) (Fig. 9).

#### Polarization of macrophages

The polarization of macrophages occurs in a wide range, from the “classically activated” M1 type to the “bypass-activated” M2 type. M1 and M2 are known as two representative and typical phenotypes that can secrete various cytokines in different tissue microenvironments. M1-type macrophages mainly secrete pro-inflammatory factors, while M2-type mainly secrete anti-inflammatory ones. The markers of the two polarization phenotypes are different, such as tumor necrosis factor-α (TNF-α) and iNOS for M1 phenotypes, and Arg1, interleukin-1ra (IL-1ra), and differentiated antigen cluster-206 (CD206) for M2 cells.

**Fig. 10:**
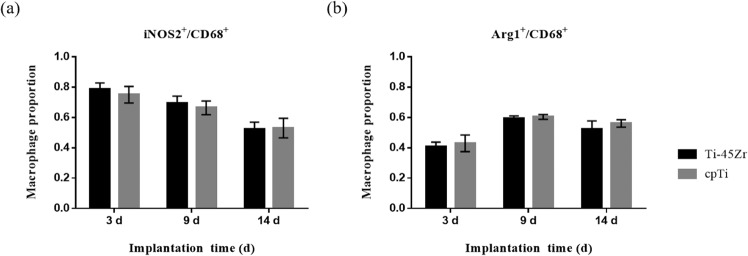
**a** The proportion iNOS2^+^ cells and CD68^+^ cells at 3, 9, and 14 days after implantation; **b** The proportion Arg1^+^ cells and CD68^+^ cells at 3, 9, and 14 days after implantation

In the present study, the iNOS2 and Arg1 markers on the surface of M1 and M2 macrophages, respectively, were labeled by immunohistochemical staining to evaluate the polarization of macrophages. Image-Pro Plus 6.0 Image analysis system was used to analyze the IHC staining images, and the ratio of iNOS2^+^ cells and Arg1^+^ cells to the number of CD68^+^ cells was calculated to evaluate the polarization conversion of M1 and M2 macrophages (Fig. 10). On the 3rd day, at the inflammation initial stage, the proportion of M1 macrophages was relatively high (*p* < 0.05), but it gradually decreased with the time extension (Fig. 10a). However, the proportion of M2 macrophages was the lowest on the 3rd day and reached the highest on the 9th day (*p* < 0.05), followed by a slight downregulation (Fig. 10b). On the 14th day, M1 and M2 macrophages showed moderate expression, and the M1/M2 polarization ratio tended to be balanced. The M1/M2 polarization was similar in the cpTi and Ti-45Zr implant groups.

### Osseointegration in vivo

#### Systemic toxicity evaluation of the Ti-45Zr alloy

**Fig. 11:**
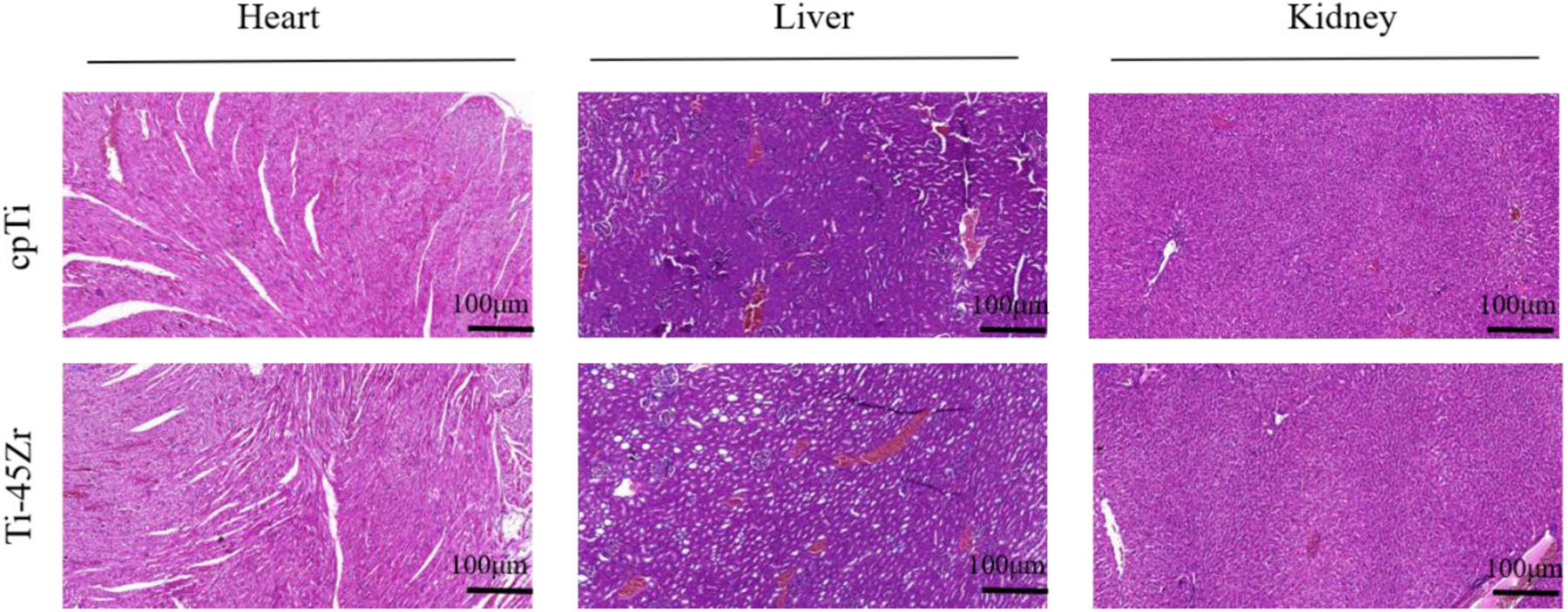
HE staining of heart, liver, and kidney of rabbits

At 16 weeks, animals were sacrificed, immediately dissected, and the main organs (liver, heart, and kidney) were removed, placed in 4% neutral buffered formalin solution, fixed for 48 h, dehydrated, and embedded. Pathological sections were stained with HE (Fig. 11). No pathological changes (ex. deformation, necrosis, or atrophy) were observed in the main organs (liver, heart, and kidney). The cell morphology of tissues was normal, and no inflammatory or apoptotic cells were observed, indicating that the Ti-45Zr alloy did not release toxic ions in vivo. Therefore, no clear toxic or side effects were observed on the main organs.

#### Gross sample observation

At the 12th and 16th weeks, animals were sacrificed for dissection and the bone segments with implant materials were removed. The wound healing of the implantation site in each group was good, the muscle color was normal and elastic, and there was no redness, no inflammatory exudate, and no scar tissue proliferation. The muscle was gradually stripped and the femur was fully exposed. The soft tissues of the four groups were normal, without inflammatory secretions. The implanted materials were tightly embedded in the bone tissue without loosening or propulsion, and no bone resorption or destruction was observed with the naked eye.

#### X-ray evaluation

**Fig. 12:**
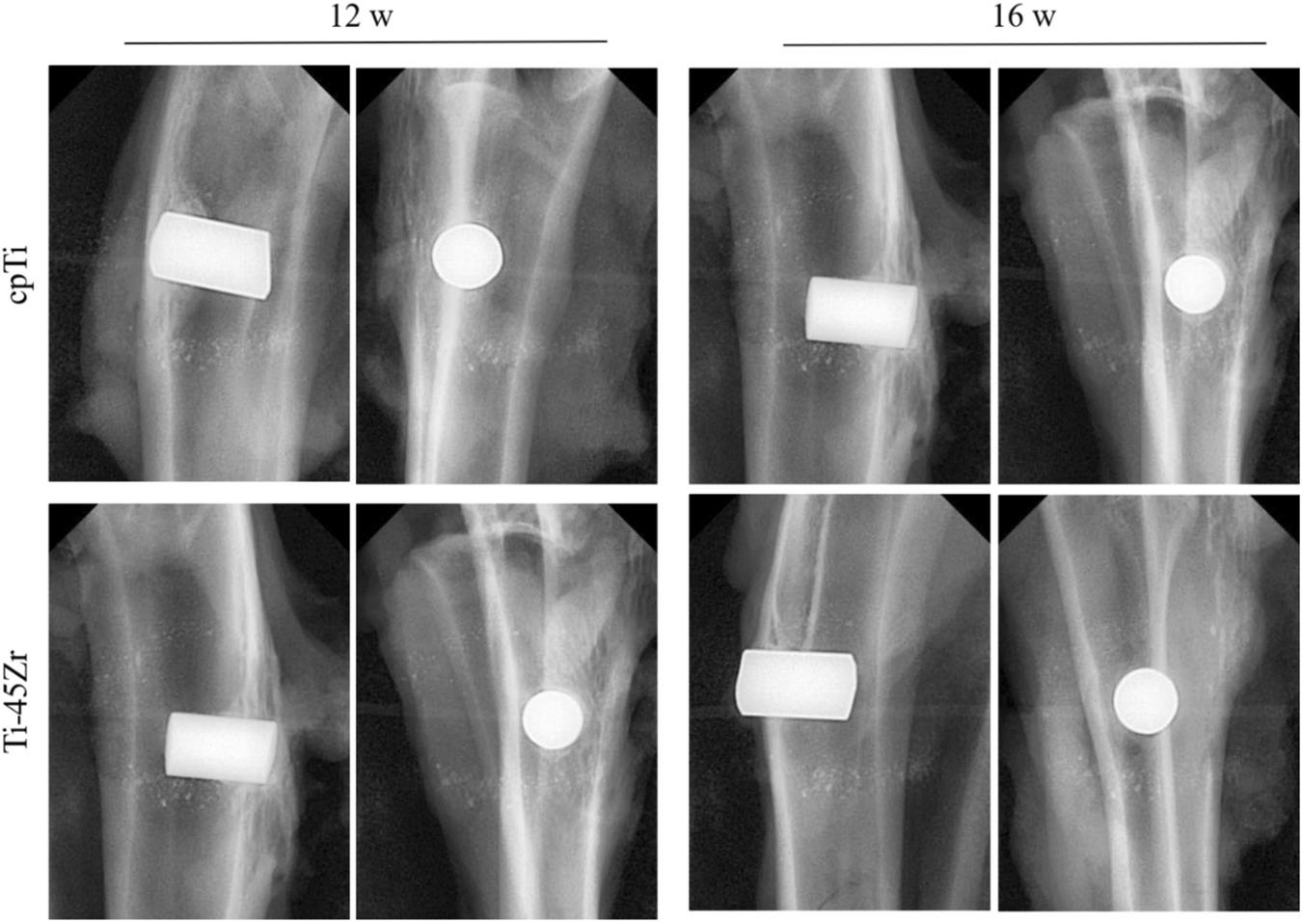
Anteroposterior and lateral radiographs of rabbit femoral specimens with implant material

Furthermore, X-ray examinations of the femurs with the implant were performed. Femur’s anteroposterior and lateral radiographs (12 and 16 w) are shown in Fig. 12. At the 12th week after surgery, the X-ray films showed that there was no bone destruction or absorption around the Ti-45Zr alloy. On the 16th postoperative week, there was no obvious low-density dark area around the implant, while white bone lines could be seen in some areas, indicating increased bone density. Hence, the implant material was closely combined with the surrounding bone tissue.

#### Hard tissue slice histomorphological analysis

**Fig. 13:**
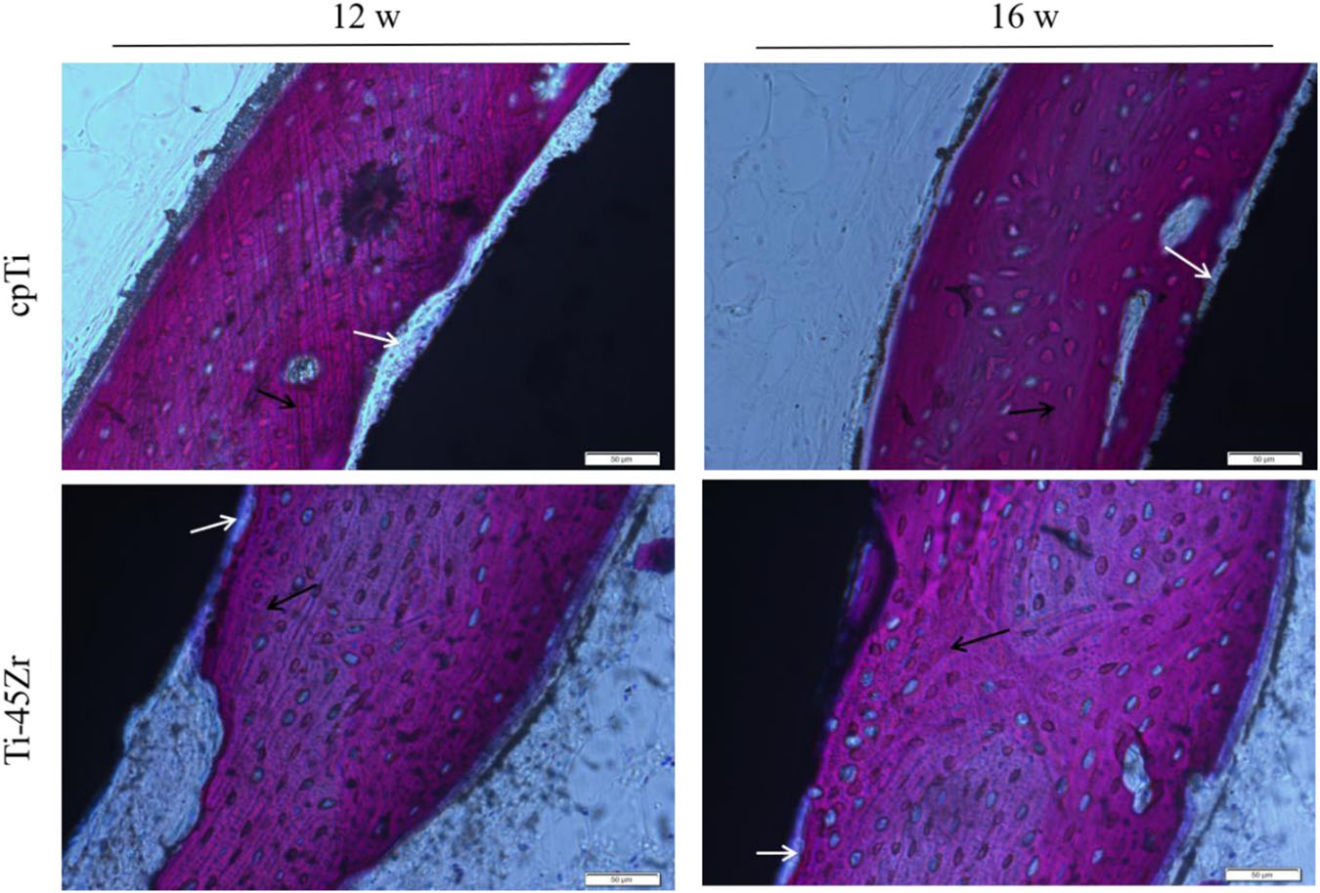
Histological signs of bone formation around the implant surface in rabbits stained with methylene blue-basic magenta: bone-binding interface (white arrow); new bone tissue (black arrow)

The bone tissue with the implant was sectioned and stained to observe the interface between the material and the bone tissue. After 12 weeks of healing, new bone was formed around all implants, while a small amount of osteoid could be seen at the bone-implant interface without significant fibrous tissue (Fig. 13). Sixteen weeks later, the amount of new bone formed around the implant increased. The new bone was in close and direct contact with the implant surface.

**Fig. 14:**
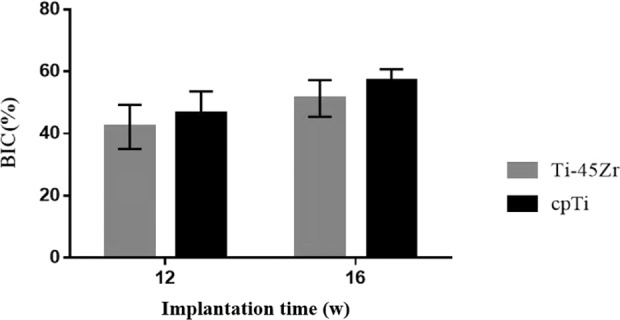
Comparison of bone-to-implant contact (BIC) percentages (100%)

The OsteoMeasure software was used to perform bone morphometric analysis on non-decalcified hard tissue grinding plates, mainly analyzing the bone to implant contact percentages (BIC). The BIC of the two groups (Ti-45Zr and cpTi) increased in a time-dependent manner (Fig. 14). There was no difference in the amount of new bone formed around Ti-45Zr and cpTi implants at 12 and 16 w after implantation.

## Discussion

The hemolysis test results confirmed that the Ti-45Zr alloy has good blood compatibility and no body side effects, consistent with the systemic short-term toxicity test results. In the systemic toxicity test, no toxic reactions, no abnormalities in the important organs, and no obvious pathological changes in tissue sections were observed in the animals. This indicates that the Ti-45Zr alloy does not have a systemic toxic reaction, confirming that Zr and Ti are non-toxic metals, and both Zr and Ti are life-reactive metals with high biosecurity.

Further, the Ti-45Zr alloy immune reaction was analyzed in vivo. The immune system is closely related to the skeletal system, and they share many cytokines, receptors, signaling molecules, and transcription factors [14]. Immune cells play a key role in bone homeostasis. Dental implants are implanted into bone tissue through invasive surgery. As a foreign body, the implant is recognized by the immune system and triggers a significant immune response that affects the behavior of bone cells. The immune response outcome will ultimately determine the fate of the implanted material in vivo [15]. Therefore, the immune response is a key factor to evaluate the stability of bone-implant materials. The immune response effects on bone integration ability of bone biomaterials is a “double-edged sword”. A favorable immune response can produce a local microenvironment that promotes osteogenesis, while an inappropriate one can lead to chronic inflammation and fibrous encapsulation around the implant [16]. The polarization of macrophages and their anti-inflammatory and pro-inflammatory properties play an important role in host response after material implantation and have a significant impact on the outcome and long-term stability of osteosseointegration [17].

In the present study, HE staining and serum biochemical experiments were used to analyze the Ti-45Zr alloy inflammatory response after implantation in vivo. On the 3rd day after implantation, the body was in an acute inflammation period, with infiltration of mainly neutrophils and lymphocytes, and mononuclear cells (HE dyeing - Fig. 7). Around implanted holes, the infiltration of macrophages was low in the beginning but with time extension the number of macrophages gradually increased and had a tendency to migrate to the material surface. On the 9th day, the number of macrophages was significantly higher than on day 3, indicating that the immune response of the organism enhanced with time. On the 14th day, there was the formation of new bone around all the implant holes, indicating that the inflammation was progressing well. Inflammation-related factors also increased with time in serum. On the 3rd day, IL-6 was significantly higher than IL-10. On the 9th day, both IL-6 and IL-10 were highly expressed, while downregulated on day 14, following the normal course of inflammation. IL-6 is a multifunctional proinflammatory cytokine secreted by M1 macrophages, involved in bone metabolism inflammation, and regulation. Therefore, it plays an important role in the bone-material interface. IL-6 can induce RANKL expression and indirectly promote osteoclast generation and activation by the RANKL/RANK OPG system. Additionally, IL-6 is essential for the early stages of bone tissue healing, and loss of IL-6 delays bone tissue mineralization and remodeling [18, 19]. IL-10 is an inflammatory cytokine mainly produced by activated M2 macrophages and, to a lesser extent, T cells. IL-10 supports BMSC homing, proliferation, and osteogenic differentiation [20]. It can also inhibit the release of various inflammatory factors and has the potential to inhibit antigen presentation. Therefore, IL-10 inhibits immune enhancement and inflammation. Recent studies have shown that IL-10 can inhibit osteoclasts formation, thereby inhibiting bone destruction and resorption occurrence [21]. Overall, the moderate expression of IL-6 and IL-10 during the inflammatory phase is beneficial to new bone formation.

Immune cells play a key role in bone homeostasis. This can be achieved through the release of cytokines that regulate osteogenesis, inducing, or inhibiting, bone formation. Macrophages are crucial to efficient osteoblast differentiation, and in vivo, macrophage depletion results in complete loss of osteoblast-mediated bone formation [22, 23]. The effect of implantable materials on the polarization of macrophages directly determines the osteointegration effect and long-term stability. Here, the IHC staining reflected the polarization of macrophages (Figs. 8 and 9). On day 3, M1 macrophages were predominant around the implant material, and on day 9, the M2 macrophage polarization ratio increased, indicating macrophage polarization phenotype transformation. Spiller pointed out that macrophages can have a short span of 3 days when M2 macrophages were completely differentiated into M1 macrophages and vice versa [24], consistent with the current results. On the 14th day, the polarization ratio of M1 and M2 macrophages was downregulated. M1 macrophages might play a key role in the early and middle stages of osteogenesis, while M2 macrophages might contribute to matrix late mineralization. Moderate conversion from the M1 to the M2 phenotype can be critical for implant materials fracture healing and osteointegration [25]. A moderate transition from the pro-inflammatory M1 phenotype to the immunoregulatory, or anti-inflammatory, M2 phenotype can be an important aspect of the bone healing process promotion, leading to a functional recovery rather than scar tissue formation [26–28]. In the present study, on the 3rd day, the early stage of inflammation presented mainly M1 macrophages (high proportion), while on the 14th day, the M2 type was dominant (moderate proportion) (Fig. 10). This indicated that the Ti-45Zr alloy conduced the balance of macrophage polarization, new bone formation, and maintenance of long-term stability.

New Zealand white rabbits were used as animal models, and bone integration stability and ability were determined by in vivo implantation experiments. Satisfactory osseointegration between the host bone and the implant material is crucial for the long-term stability of dental implants. After 12 w of healing, new bone was formed around the implant, while a small amount of osteoid could be seen at the bone-implant interface without clear fibrous tissue (Fig. 13). There was a certain gap between the bone tissue and the implant interface, in which osteoblasts could be seen. With implantation time prolongation, new bone formation increased around all implants until 16 w, and the space between the new bone and the implant material surface decreased, leading to close and direct contact. There were no blank non-contact images and low-density bone absorption images between Ti-45Zr alloy and bone tissue (Fig. 12). The results showed that the Ti-45Zr alloy had direct contact with bone tissue, and there was no obvious fibrous layer formed between the alloy and bone tissue. The osteointegration ability of the implant material is related to many factors, including alloying elements, surface properties, elastic modulus, porosity, and pore size. In the current study, the Ti-45Zr alloy presented good osseointegration ability, which might be attributed to its excellent mechanical properties (low elastic modulus). Additionally, zirconium (Zr) and titanium (Ti) are metals with good osseointegration activity. After six months of Zr and Ti threaded implantation into the oral alveolar bone of pigs, Kulakov et al. confirmed that the amount of new bone formation around Zr implants was higher than that around Ti implants under the same conditions [29]. The success and peri-implant bone absorption rates of the Ti-Zr narrow-diameter implant were comparable to that of cpTi implants of the same size [30].

Overall, the Ti-45Zr alloy has superior biocompatibility and conducted the balance of macrophage polarization. Particularly, Ti-45Zr showed good osteogenic activity in vivo, being a promising alternative to cpTi as a dental implant. However, this in vivo study did not provide a quantitative analysis of bone formation around the implant, which requires further investigation.

## Conclusion

The Ti-45Zr alloy showed good blood compatibility and had no toxic side effects in vivo. With the extension of implantation time, the inflammation recovery was satisfactory and new bone formation was observed around the material. Also, the Ti-45Zr alloy was beneficial to the balance of macrophage polarization. Meanwhile, the in vivo analyses demonstrated that the Ti-45Zr had good osteogenic activity. Altogether, these results suggested that the Ti-45Zr showed excellent biocompatibility, immunoregulatory ability, and osteointegration to support bone formation, being a good prospect for the transformation and application of dental implant materials.

## References

[1] Peng KH, Zhou YP, Dai YH (2021). The effect of denture restoration and dental implant restoration in the treatment of dentition defect: a systematic review and meta-analysis. Ann Palliat Med.

[2] Domingo JL (2002). Vanadium and tungsten derivatives as antidiabetic agents-A review of their toxic effects. Biol Trace Elem Res.

[3] Wang X, Jiang M, Zhou ZW, Gou JH (2017). 3D printing of polymer matrix composites: a review and prospective. Compos Part B Eng.

[4] Zhang XZ, Leary M, Tang HP (2018). Selective electron beam manufactured Ti-6Al-4V lattice structures for orthopedic implant applications: current status and outstanding challenges. Curr Opin Solid State Mater Sci..

[5] Brizuela A, Herrero-Climent M, Rios-Carrasco E (2019). Influence of the elastic modulus on the osseointegration of dental implants. Materials.

[6] Oliveira NTC, Biaggio SR, Rocha-Filho RC (2002). Studies on the stability of anodic oxides on zirconium biocompatible alloys. J Braz Chem Soc.

[7] Saldana L, Mendezvilas A, Jiang L (2007). In vitro biocompatibility of an ultrafine grained zirconium. Biomaterials.

[8] Mehjabeen A, Song T, Xu W (2018). Zirconium alloys for orthopaedic and dental applications. Adv Eng Mater.

[9] Correa DRN, Vicente FB, Donato TAG (2014). The effect of the solute on the structure, selected mechanical properties, and biocompatibility of Ti-Zr system alloys for dental application. Mater Sci Eng C.

[10] Iegami CM, Uehara PN, Sesma N (2017). Survival rate of Ti-Zr narrow diameter dental implants versus commercially pure diameter dental implants versus commercially pure titanium diameter dental implant. Clin Implant Dent Relat Res.

[11] Daniela I, Cristian P, Andrei B (2020). The trends of Ti-Zr alloy research as a viable alternative for Ti and Ti-16Zr roxolid dental implants. Coatings.

[12] Pinghua O, Cong H, Jue L (2021). Cytocompatibility of Ti-xZr alloys as dental implant materials. J Mater Sci Mater Med.

[13] Papaspyridakos P, Chen CJ, Singh M (2012). Success criteria in implant dentistry: a systematic review. J Dent Res.

[14] Albrektsson T, Chrcanovic B, Jacobsson M (2017). Osseointegration of implants-a biological and clinical overview. JSM Dent Sur.

[15] Xuxi C, Lin ZH, Dong W. The effects of Titanium surfaces modified with an antimicrobial peptide GL13K by silanization on polarization, anti- inflammatory, and proinflammatory properties of macrophages. BioMed Res Int. 2020; 10.1155/2020/2327034. 10.1155/2020/2327034 PMC7396038 32775410

[16] Pajarinen J, Lin T, Gibon E (2019). Mesenchymal stem cell-macrophage crosstalk bone healing. Biomaterials.

[17] Mengchi X, Dong Z, Jiang C (2014). In vitro assessment of three- dimensionally plotted nagelschmidtite bioceramic scaffolds with varied macropore morphologies. Biomaterials.

[18] Yang X, Ricciardi BF, Hernandez-Soria A (2007). Callus mineralization and Maturation are delayed during fracture healing in interleukin-6 knockout mice. Bone.

[19] Bryan NB, Buddy DR, Stuart BG (2012). Macrophage polarization: an opportunity for improved outcomes in biomaterials and regenerative medicine. Biomaterials.

[20] Klopfleisch R (2016). Macrophage reaction against biomaterials in the mouse model-Phenotypes, functions and markers. Acta Biomater.

[21] Mohamed SGK, Sugiyama E, Shinoda K (2007). Interleukin-10 inhibits RANKL-mediated expression of NFATcl in part via suppression of c-Fos and c-Jun in RAW264.7 cells and mouse bone marrow cells. Bone.

[22] Sinder BP, Pettit AR, McCauley LK (2015). Macrophages: their emerging roles in bone. J Bone Min Res.

[23] Xin W, Yu L, Yuan F (2020). The role of macrophages in osseointegration of dental implants: An experimental study in vivo. J Biomed Mater Res.

[24] Spiller KL, Nassiri S, Witherel CE (2015). Sequential delivery of immunomodulatory cytokines to facilitate the M1-to-M2 transition of macrophages and enhance vascularization of bone scaffolds. Biomaterials.

[25] Kara LS, Sina N, Claire EW (2015). Sequential delivery of immunomodulatory cytokines to facilitate the M1-to-M2 transition of macrophages and enhance vascularization of bone scaffolds. Biomaterials.

[26] Brown BN, Londono R, Tottey S (2012). Macrophage phenotype as a predictor of constructive remodeling following the implantation of biologically derived surgical mesh materials. Acta Biomater.

[27] Sussman EM, Halpin MC, Muster J (2014). Porous implants modulate healing and induce shifts in local macrophage polarization in the foreign body reaction. Ann Biomed Eng.

[28] Blatt SE, Lurier EB, Risser GE (2020). Characterizing the macrophage response to immunomodulatory biomaterials through gene set analyses. Tissue Eng.

[29] Kulakov OB, Doktorov AA, Diakova SV (2015). Experimental study of osseointegration of zirconium and titanium dental implants. Morfologiia.

[30] Iegami CM, Uehara PN, Sesma N (2017). Survival rate of Ti-Zr narrow diameter dental implants versus commercially pure diameter dental implants versus commercially pure titanium diameter dental implants. Clin Implant Dent Relat Res.

